# Hypoxia and Prostaglandin E Receptor 4 Signalling Pathways Synergise to Promote Endometrial Adenocarcinoma Cell Proliferation and Tumour Growth

**DOI:** 10.1371/journal.pone.0019209

**Published:** 2011-05-12

**Authors:** Rob D. Catalano, Martin R. Wilson, Sheila C. Boddy, Andrew T. M. McKinlay, Kurt J. Sales, Henry N. Jabbour

**Affiliations:** Medical Research Council Human Reproductive Sciences Unit, The Queen's Medical Research Institute, University of Edinburgh, Edinburgh, United Kingdom; Beth Israel Deaconess Medical Center, United States of America

## Abstract

The prostaglandin endoperoxide synthase (PTGS) pathway is a potent driver of tumour development in humans by enhancing the biosynthesis and signalling of prostaglandin (PG) E_2_. PTGS2 expression and PGE_2_ biosynthesis is elevated in endometrial adenocarcinoma, however the mechanism whereby PTGS and PGE_2_ regulate endometrial tumour growth is unknown. Here we investigated (a) the expression profile of the PGE synthase enzymes (PTGES, PTGES-2, PTGES-3) and PGE receptors (PTGER1–4) in endometrial adenocarcinomas compared with normal endometrium and (b) the role of PTGER4 in endometrial tumorigenesis in vivo. We found elevated expression of PTGES2 and PTGER4 and suppression of PTGER1 and PTGER3 in endometrial adenocarcinomas compared with normal endometrium. Using WT Ishikawa endometrial adenocarcinoma cells and Ishikawa cells stably transfected with the full length PTGER4 cDNA (PTGER4 cells) xenografted in the dorsal flanks of nude mice, we show that PTGER4 rapidly and significantly enhances tumour growth rate. Coincident with enhanced PTGER4-mediated tumour growth we found elevated expression of PTGS2 in PTGER4 xenografts compared with WT xenografts. Furthermore we found that the augmented growth rate of the PTGER4 xenografts was not due to enhanced angiogenesis, but regulated by an increased proliferation index and hypoxia. In vitro, we found that PGE_2_ and hypoxia independently induce expression of PTGER4 indicating two independent pathways regulating prostanoid receptor expression. Finally we have shown that PGE_2_ and hypoxia synergise to promote cellular proliferation of endometrial adenocarcinoma cells.

## Introduction

Endometrioid adenocarcinoma arising from the endometrial glandular epithelium is one of the leading causes of cancer-related morbidity in women world-wide [1], [2]. The onset of the disease occurs more frequently post menopause and is associated with a host of risk factors, including high levels of unopposed estrogen, endometrial hyperplasia and obesity [1].

Although the molecular mechanisms responsible for the initiation of the disease are multifactorial, there is now much evidence to substantiate genetic alterations and the inactivation of tumour suppressor genes in the neoplastic transformation of cells to facilitate uncontrolled proliferation and tumour growth [3]. Indeed one of the most common mutations in endometrial adenocarcinomas is located in the dual specificity phosphatase PTEN (phosphatase and tensin homologue deleted from chromosome ten) gene [2]. Animal models in which PTEN expression is inactivated in the endometrium have shown that loss of the PTEN function rapidly and unfailingly induces endometrial cancer [4]. Inactivation of PTEN function promotes protein kinase B (also called Akt) activation which in turn stimulates the activity of several target genes involved in cell proliferation [5]. One the major targets of Akt signalling in endometrial adenocarcinoma cells, is prostaglandin endoperoxide synthase (PTGS) 2 (also called cyclooxygenase 2) [4], [6].

PTGS2 converts arachidonic acid, the rate limiting substrate in the prostaglandin biosynthesis cascade to the unstable intermediate prostaglandin (PG)G_2_ which is subsequently converted to PGH_2_ [7]. In turn PGH_2_ is metabolised by terminal prostaglandin synthase enzymes, which are named according to the prostaglandin they biosynthesise. For example prostaglandin E synthase (PTGES) enzymes, of which there are three isoforms (PTGES, PTGES-2, PTGES-3) produce PGE_2_ [8]. PTGS2 expression is elevated in numerous neoplastic diseases including endometrial adenocarcinomas resulting in elevated biosynthesis of PGE_2_ [9], [10]. In vitro and in vivo model systems overexpressing PTGS2 have shown that PGE_2_ can promote tumorigenesis by altering cell cycle progression, inhibiting apoptosis and enhancing cellular proliferation [11], [12], [13]. Furthermore PGE_2_ can promote tumour progression by down-regulating tumour suppressor genes such as tuberous sclerosis-2 (TSC2) [14], P53 and retinoblastoma in epithelial cells [11].

Following biosynthesis, PGE_2_ is transported out of the cell where it binds to and activates PGE_2_ G protein-coupled receptors of which there are four isoforms PTGER 1–4 [15]. PTGER4 expression is elevated in numerous cancers where it can transduce pathways important for cancer cell survival and tumour progression, such as the Akt, extracellular signal regulated kinase (ERK1/2) and early growth response factor-1 [16], [17], [18]. The aim of this study was to identify the components of the PTGS-PGE_2_ pathway in endometrial adenocarcinomas which regulate tumour growth.

Here, we show suppression of PTGER1 and PTGER3 and elevated expression of PTGES2 and PTGER4 in endometrial adenocarcinomas compared with normal endometrium. We show that inoculation of nude mice with Ishikawa endometrial adenocarcinoma cells stably expressing PTGER4 promotes formation of tumour xenografts which grow at a significantly faster rate compared with tumours arising from WT control cells. Moreover these tumours displayed elevated expression of PTGS2 compared with tumours arising from WT Ishikawa cells. Morphometric and stereological analyses showed no difference in blood vessel density or VEGF expression in PTGER4 tumours compared with WT tumours indicating that PTGER4-mediated tumour growth was not a result of increased angiogenesis. The PTGER4 tumours displayed an increase in cellular proliferation and increased areas of hypoxia. Using in vitro models, we subsequently demonstrated that hypoxia and PGE_2_ synergise to induce expression of PTGER4 leading to increased cellular proliferation.

## Materials and Methods

### Ethics Statement

Ethical approval was obtained from Lothian Research Ethics Committee (LREC/07/S1103/32, LREC/05/S1103/29 and LREC/1999/6/4) and written informed consent was obtained from all subjects before tissue collection. All animal care and experimental protocols were approved by the animal ethics committee of the University of Edinburgh and the Home Office of the United Kingdom government under the guidelines of the Animals (Scientific Procedures) Act 1986.

### Human tissue

Poorly (grade 3; n = 10), moderately (grade 2; n = 10) and well (grade 1; n = 9) differentiated endometrial adenocarcinoma tissues with clinical characteristics as described in Table 1 [19] were collected from women undergoing hysterectomy. All women had been pre-diagnosed on endometrial biopsy to have endometrial adenocarcinoma of the endometrioid type. The diagnosis of endometrial adenocarcinoma was confirmed histologically in all cases as defined in Table 1 and the percentage of tumour cells to stroma was estimated to be not less than 75%∶25%. Normal endometrium with clinical parameters as outlined in Table 2 from the proliferative (n = 10), early secretory (n = 10), mid secretory (n = 10) and late secretory (n = 10) phases of the menstrual cycle, was collected from women undergoing surgery for gynecological procedures in whom histological examination of endometrium was normal with no underlying endometrial pathology (Pipelle, Laboratoire CCD, France). All biopsies were dated according to stated last menstrual period (LMP) and confirmed by hormone analysis and histological assessment according to the criteria of Noyes *et al.* [20].

**Table 1:** Clinical parameters and tumour characteristics for well (n = 10), moderately (Mod, n = 10) and poorly differentiated (n = 10) endometrial adenocarcinomas.

SAMPLE NO	GRADE	FIGO STAGE
1	Well	Ib
2	Well	Ib
3	Well	Ia
4	Well	Ic
5	Well	IIIa
6	Well	Ib
7	Well	Ib
8	Well	Ib
9	Well	Ia
10	Mod	Ic
11	Mod	Ic
12	Mod	Ia
13	Mod	Ib
14	Mod	IIb
15	Mod	Ib
16	Mod	Ic
17	Mod	IIb*
18	Mod	IIIa
19	Mod	Ib
20	Poor	Ia
21	Poor	IIIa
22	Poor	Ib
23	Poor	Ib
24	Poor	IIIa
25	Poor	Ib
26	Poor	Ib
27	Poor	IIIa
28	Poor	IIIa
29	Poor	IIb*

All endometrial adenocarcinomas studied were of pure or predominantly of endometrioid type. There were no pure serous or clear cell subtypes in the series and none of the tumours showed carcinosarcoma.

*in these two samples myometrial invasion was <50%.

**Table 2:** Clinical parameters for normal endometrial samples from proliferative phase, early secretory, mid secretory and late secretory phase endometrium (n = 10).

SAMPLE NO	HISTOLOGY	PROGESTERONE nmol/L	ESTRADIOL pmol/L
1	Proliferative	4.26	339.38
2	Proliferative	2.22	641.37
3	Proliferative	4.62	525.00
4	Proliferative	4.57	495.00
5	Proliferative	2.82	214.00
6	Proliferative	1.78	400.00
7	Proliferative	4.80	204.00
8	Proliferative	3.12	731.00
9	Proliferative	2.32	1796.00
10	Proliferative	0.17	989.70
11	Early Secretory	63	353
12	Early Secretory	16.55	556
13	Early Secretory	63	458
14	Early Secretory	93.15	848
15	Early Secretory	9.03	90.43
16	Early Secretory	11.58	75.99
17	Early Secretory	10.371	876.16
18	Early Secretory	38.48	243.08
19	Early Secretory	26.74	768.92
20	Early Secretory	101.1	584
21	Mid Secretory	33.29	242.72
22	Mid Secretory	36.94	280.35
23	Mid Secretory	4.54	544.04
24	Mid Secretory	79.8	748
25	Mid Secretory	32.67	277
26	Mid Secretory	25.74	292
27	Mid Secretory	37.54	658
28	Mid Secretory	36.17	294.79
29	Mid Secretory	14.53	392
30	Mid Secretory	9.78	248.03
31	Late Secretory	0.47	0
32	Late Secretory	6.60	174.75
33	Late Secretory	8.36	326
34	Late Secretory	65.65	1075
35	Late Secretory	133.6	876
36	Late Secretory	3.85	55
37	Late Secretory	7.67	248.89
38	Late Secretory	20	410.91
39	Late Secretory	8.36	326
40	Late Secretory	65	1075

### Cell culture

Wild-type (WT) Ishikawa cells (European Collection of Cell Culture, Wiltshire, UK) were maintained as described previously [14]. Stable PTGER4 transfectant cells (PTGER4 cells) were maintained under the same conditions with the addition of a maintenance dose of 200 µg/ml G418 (Calbiochem, UK).

### PTGER4 amplification and Cell Transfections

The PTGER4 receptor cDNA clone and stable cell lines were constructed by Cytomix Ltd (Cambridge, UK). PTGER4 cDNA (GenBank Accession N0. D28472) was amplified by polymerase chain reaction (PCR) and the product was directionally cloned into the mammalian expression vector gWIZ3.0 (Gene Therapy Systems, Cambridge, UK) at the *Hind III* and *Bam H1* restriction sites and the orientation of the construct confirmed by automated DNA sequencing. The PTGER4 cDNA was transfected into Ishikawa cells and individual cell populations were selected for with addition of 800 µg/ml G418. Full selection was confirmed by the 100% death of non-transfected control cells and five single PTGER4 sense clones were selected and expanded for Western blot analysis. The clones with the highest level of PTGER4 expression were expanded and stored in liquid nitrogen. Based on the relative quantification of PTGER4 expression, two sense clones (S20 and S22) exhibiting similar phenotypic and biochemical alterations were supplied. The results of our studies using the S22 clone are presented here.

#### cAMP Assay

PGE_2_ induced cAMP accumulation was determined by seeding 2×10^5^ WT or PTGER4 cells/well in 6-well plates. The cells were serum-starved in the presence of 3 µg/ml of indomethacin. Thereafter, the cells were pre-treated with the phosphodiesterase inhibitor 3-isobutyl-1-methyl xanthine (IBMX; Sigma, Dorset, UK) to a final concentration of 0.2 mM in serum-free medium for 30 min. Cells were treated for 5 min with 100 nM PGE_2_, or left unstimulated (time 0). After incubation the cells were lysed in R&D Cell Lysis Buffer™ (R&D Systems, Oxford UK) and cAMP release was determined by ELISA using cAMP Kit (R&D Systems) according to manufacturer's protocol. Data are represented as mean ± SEM of three independent experiments.

#### Xenograft Experiments

WT or PTGER4 cells (2.5×10^6^) were subcutaneously injected in a volume of 100 µl into the left and right flanks of ten CD1-*Foxn1^nu^* mice (Charles River, Tranent, UK). All animals were housed in individually ventilated cages.

The resulting tumours (n = 20 WT tumours and n = 20 PTGER4 tumours) were measured twice weekly by digital calipers. Tumour volume was calculated using the formula; Volume = 0.5×(vertical measurement/10)×(horizontal measurement/10)×(horizontal measurement/10). After a period of 35 days, the animals were sacrificed. Two hours prior to sacrifice, the animals received an intraperitoneal (i.p) injection of 50 mg/kg of 5-Bromo-deoxy Uridine (BrdU, Sigma, UK). One hour prior to sacrifice, the animals received an i.p injection of 60 mg/kg of Hypoxyprobe (pimonidazole hydrochloride; HPI, Inc, Burlington, MA, USA). The tumours were removed and half of each was placed in 4% neutral buffered formalin (NBF, Sigma, UK) and a quarter section was snap-frozen in QIAzol (Qiagen, Crawley, UK) for RNA extraction.

#### Quantitative RT-PCR

RNA from the tumours was extracted using the RNeasy Mini Kit (Qiagen). RNA samples were reverse-transcribed using VILO (Invitrogen, Paisley, UK) according to the manufacturer's guidelines. RT-PCR analysis of the genes of interest was carried out using an ABI Prism 7500 (Applied Biosystems, Warrington, UK). Primer and FAM (6-carboxyfluorescein)-labelled probe sequences are shown in Table 3. For PTGES3, primer only amplification products were detected using SYBR™ Green (Applied Biosystems) incorporation during the PCR reaction. Gene expression was normalised to 18S ribosomal RNA (Applied Biosystems) as an internal standard. Results are expressed relative to a standard (pooled normal human endometrial tissue cDNA) included in all reactions. Data are represented as mean ± SEM.

**Table 3:** Sequences of Taqman primers and probe.

Gene Symbol	Position	Nucleotide Sequence (5′-3′)
HIF-1α	Forward	CGC ATC TTG G ATA AGG CCT CTG T
	Reverse	AAT CAC CAG G CAT CCA GAA GTT TC
PTGER1	Forward	AGA TGG TGG GCC AGC TTG T
	Reverse	GCC ACC AAC ACC AGC ATT G
PTGER2	Forward	GAC CGC TTA CCT GCA GCT GTA C
	Reverse	TGA AGT TGC AGG CGA GCA
PTGER3	Forward	GAC GGC CAT TCA GCT TAT GG
	Reverse	TTG AAG ATC ATT TTC AAC ATC ATT ATC A
PTGER4	Forward	ACG CCG CCT T ACT CCT ACA TG
	Reverse	AGA GGA CGGG TGG CGA GAA T
PTGES	Forward	TCA ACT GTG G ATG GCA AGA GC
	Reverse	CTA ACC TCC C TGA GCC CTC CT
PTGES1	Forward	CGG AGG CCC CCA GTA TTG
	Reverse	GGG TAG ATG GTC TCC ATG TCG TT
PTGES2	Forward	CCT CAT CAG CAA GCG ACT
	Reverse	CCA CTT GTC AGC AGC CTC A

#### Confocal Immunofluorescent microscopy

Approximately 1.0×10^4^ WT or PTGER4 cells were seeded in chamber slides and fixed in 100% ice-cold methanol. Following fixing, cells were washed in TBS (50 mM Tris-HCl, 150 mM NaCl pH 7.4) and blocked using 5% normal swine serum diluted in TBS. Subsequently, the cells were incubated with polyclonal rabbit anti-PTGER4 antibody at a dilution of 1∶50 at 4°C for 18 hours. Control cells were incubated with rabbit immunoglobulin (IgG). Thereafter, the cells were incubated with secondary swine anti-rabbit tetramethyl rhodamine isothyocyanate (TRITC; Dako Corp, High Wycombe, UK) at 25°C for 20 mins. Cells were then mounted in Permafluor (Immunotech-Coulter, Buckinghamshire, UK) and coverslipped prior to imaging.

#### Immunohistochemistry

Tumour tissue was embedded in paraffin wax and sections of 5 µm thickness of WT (n = 10) and PTGER4 (n = 10) tumours were cut and mounted on to coated slides. The sections were de-waxed and rehydrated through graded alcohols before antigen retrieval in 0.01 M citrate phosphate buffer. Immunostaining was performed using the Vision Biosystems Bond Immunostaining Robot (Leica Microsystems, Wezlar, Germany) under normal operating conditions as described in our previous study [21] using a sheep-anti-BrdU antibody (1∶6000; Fitzgerald, UK) or Hypoxyprobe mouse monoclonal antibody (Vision Biosystems mouse polymer protocol at 1∶50 dilution). For CD31, sections were blocked in normal porcine serum and incubated with rabbit-anti-CD31 (1∶250; Abcam, Cambridge, UK) overnight at 4°C. Sections were incubated with a 1∶250 dilution of swine-anti-rabbit biotinlyated secondary antibody (DAKO, Cambridge, UK). The sections were then incubated with streptavidin peroxidase (1∶1000) and detected using 3-3′ diaminobenzidine (DAB; DAKO) prior to imaging.

#### Stereological analysis

Images were captured using a ×40 plan apo objective from a BH2 microscope (Olympus, Tokyo, Japan) fitted with an automatic stage (Prior Scientific Instruments Ltd., Cambridge, UK) and video camera (HV-C20; Hitachi, Tokyo, Japan) and were analyzed with Image-Pro Plus 4.5.1 software with a Stereology 5.0 plug-in (Media Cybernetics, Wokingham, Berkshire, UK). 100 random fields of view were counted for each tumour section (n = 5 WT and PTGER4 tumour sections for Hypoxyprobe counts, n = 7 tumour sections each for BrdU counts and n = 11 tumour sections each for CD31 counts).

#### Hypoxia experiments

Ishikawa WT and PTGER4 cells were plated at a density of 2.5×10^5^ cells per well in triplicate in 6 well plates. Cells were cultured under normoxic conditions (20% oxygen) in a standard incubator at 37°C, 95% air/5% CO_2_ or placed in hypoxia chamber (1% oxygen) at 37°C, 80% N_2_/10% H_2_/10% CO_2_ for 24 hours in the presence/absence of 8.4 µM indomethacin [22]. RNA was extracted, reverse transcribed and PTGER4 expression was determined by quantitative RT-PCR as described above. In the case of hypoxic cells, these manipulations were performed inside the hypoxia chamber to avoid reoxygenation. Data are represented as mean ± SEM of four independent experiments.

#### Proliferation assay

Ishikawa WT and PTGER4 cells were plated at a density of 5.0×10^3^ cells per well in 96 well plates. The following day cells were incubated in serum free medium in the absence/presence of 8.4 µM indomethacin. Thereafter cells were treated with vehicle or 1 µM PGE_2_ in the presence/absence of indomethacin. Plates were incubated under normoxic or hypoxic conditions as described above for 24 hours. Cell proliferation was measured using the CellTitre96Aqueous One Solution Proliferation Reagent (Promega, Southampton, UK) as per the manufacturer's protocol. Data are represented as mean ± SEM of four independent experiments.

#### Statistics

Data were analysed using an unpaired t-test or a one-way ANOVA with Tukey's multiple comparison post test where applicable (GraphPad Prism, GraphPad Software Inc., USA). Box and whisker plots: Boxes represent the data lying within the 5^th^ to the 95^th^ percentile and the whiskers represent the minimum and maximum values.

## Results

### Expression profiling of the PGE_2_-PTGER signalling pathway in endometrial adenocarcinomas

**Figure 1:**
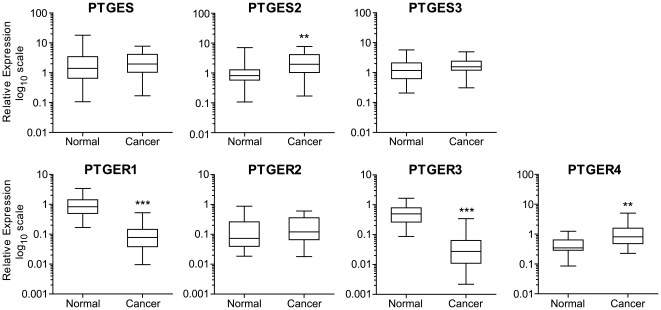
Expression profile of the PTGES-PTGER system in endometrial adenocarcinoma and normal endometrium. mRNA expression of PGE_2_ synthase isoforms (PTGES, -2, -3) and E-series prostaglandin receptors (PTGER1–4) in normal endometrium (n = 40; comprising a pool of proliferative n = 10; early secretory n = 10; mid-secretory n = 10 and late secretory n = 10 phase endometrium) and endometrial cancer (n = 29; comprising a pool of poorly differentiated n = 10, moderately differentiated n = 10 and well differentiated endometrial adenocarcinoma n = 9). **, *** represent significance at P<0.01 and P<0.001 for cancer vs normal.

We recently undertook a comprehensive analysis of the expression profile of the prostaglandin enzymes and receptors in normal endometrium across the menstrual cycle [23]. Here, we undertook a comprehensive analysis of the expression profile of the PGE synthase enzymes (PTGES, -2, -3) and E-series prostanoid receptors (PTGER1–4) in endometrial adenocarcinoma by quantitative RT-PCR analysis (Figure 1 and Figure S1). We found no significant difference in expression patterns of the synthase enzymes or prostanoid receptors between the different grades of endometrial adenocarcinoma investigated and henceforth pooled the data for analysis. We found elevated expression of PTGES2 (P<0.01) and PTGER4 (P<0.01) in all grades of endometrial adenocarcinomas compared with pooled normal endometrium. Interestingly, within our sample set, the present study revealed a significant decrease in the expression of PTGER1 (P<0.001) and PTGER3 (P<0.001), which are reported to differentially couple to Gq/11 and Gi proteins and which mobilises intracellular calcium and suppresses cAMP respectively. These results indicate a bias towards the PGE_2_-PTGER4-cAMP pathway as a potential driver of endometrial cancer.

### PTGER4 enhances growth of tumour xenografts in vivo

**Figure 2:**
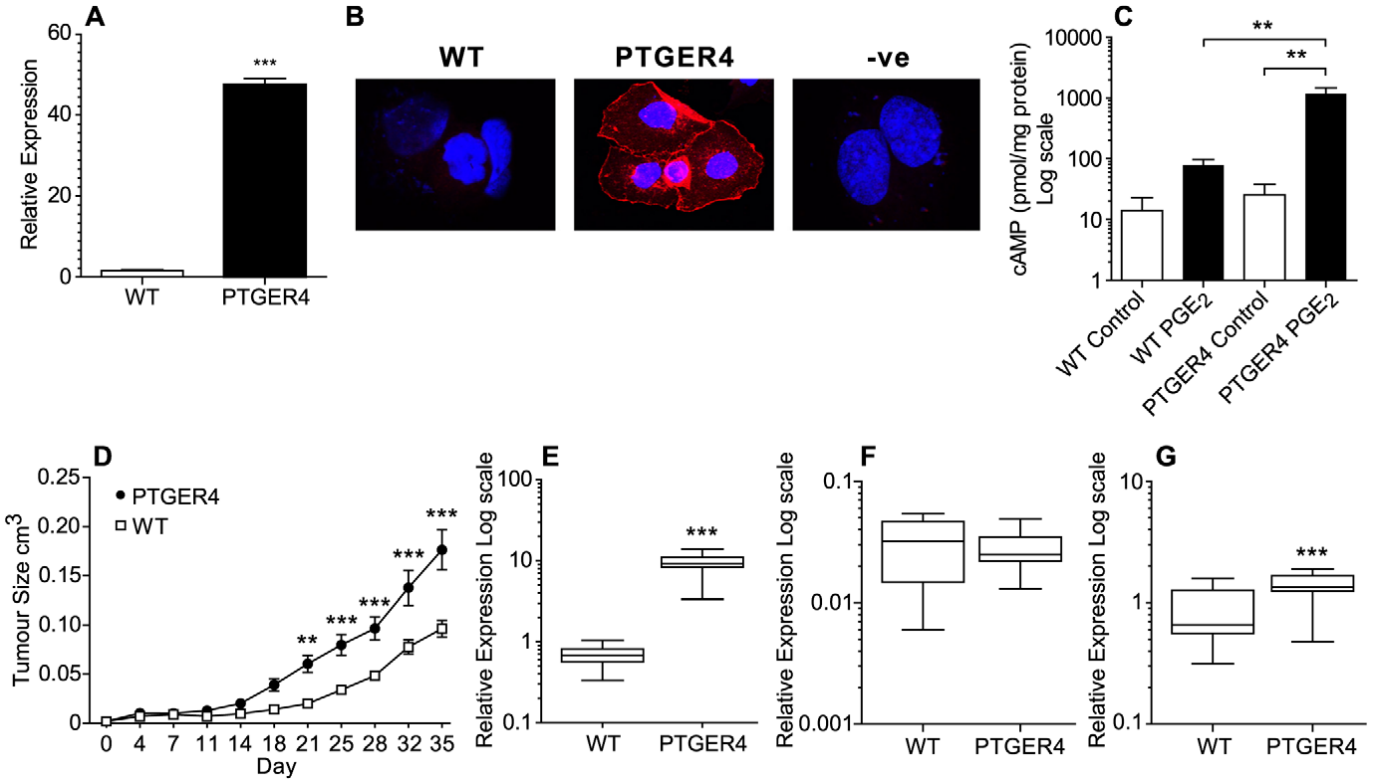
PTGER4 enhances tumour growth in a nude mouse xenograft model. (**A**) PTGER4 mRNA expression in WT Ishikawa cells compared with Ishikawa cells stably transfected with the full length PTGER4 cDNA transcript (n = 3). (**B**) Immunolocalisation of PTGER4 expression in WT and PTGER4 stable cell lines. (**C**) cAMP accummulation in WT and PTGER4 stable cell lines in response to treatment with 100 nM PGE_2_ for 0 or 5 minutes (n = 3). (**D**) Tumour growth in CD1-*Foxn1^nu^* mice following dorsal flank injection with either Ishikawa WT or PTGER4 stable cells. Tumour growth in WT (n = 20) and PTGER4 (n = 20) xenografts was monitored over a 35 day period as described in the methods. (**E**) PTGER4 (**F**) PTGS1 and (**G**) PTGS2 mRNA expression in either Ishikawa WT (n = 10) or PTGER4 (n = 10) xenograft tumours. **, *** represent significance at P<0.01 and P<0.001.

In endometrial adenocarcinomas, PTGER4 is localised to the neoplastic epithelial compartment [9]. To elucidate a role for the PGE_2_-PTGER4-cAMP pathway in endometrial tumour growth, we stably transfected Ishikawa endometrial epithelial cells with the full length PTGER4 cDNA transcript. Quantitative RT-PCR analysis showed elevated PTGER4 expression in PTGER4 cells compared with WT cells (Figure 2A, P<0.001). Using immunohistochemistry and confocal laser microscopy, we localised the site of expression of the translated PTGER4 transgene to the plasma membrane compartment (Figure 2B).

PTGER4 couples to Gs resulting in increased cAMP accumulation. We assessed functionality of the transfected PTGER4 cDNA transcript by cAMP ELISA in response to treatment with 100 nM PGE_2_ for 5 minutes. A significant increase in cAMP accumulation was observed in PTGER4 cells compared with WT cells following treatment with PGE_2_ (Figure 2C, P<0.05).

To assess the role of PTGER4 in endometrial cell tumorigenesis *in vivo*, we xenografted either WT cells or PTGER4 cells into the dorsal flanks of CD1-*Foxn1^nu^* mice and measured the resulting tumour growth over a 35 day period. We found a significant divergence in the growth rate of the tumours arising from the respective cell types, with PTGER4 xenograft tumours growing at a significantly higher rate after day 21 compared with WT xenograft tumours (Figure 2D; ** p<0.01 to *** p<0.001 comparing the volume of PTGER4 xenograft tumours versus WT xenograft tumours). We confirmed elevated PTGER4 expression levels in PTGER4 xenograft tumours compared with WT xenograft tumours by quantitative RT-PCR analysis (Figure 2E, P<0.001) indicating that expression of the transgene was maintained *in vivo*.

Since tumour growth *in vivo* has been shown to be regulated by the PTGS-PGE_2_ pathway [24], we next assessed the expression of the prostaglandin endoperoxide synthase enzymes responsible for producing PGE_2_. We found elevated expression of PTGS2 (Figure 2G, P<0.001) but not PTGS1 (Figure 2F) in PTGER4 xenograft tumours compared with WT tumours.

### Xenograft tumour growth in PTGER4 tumours is not due to enhanced angiogenesis

**Figure 3:**
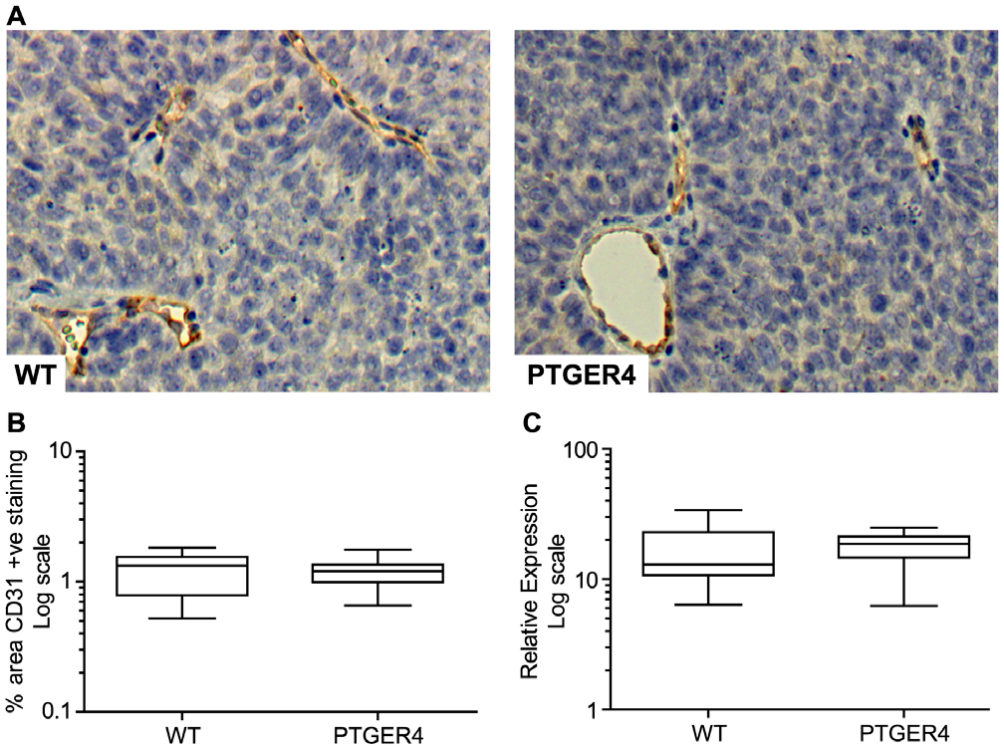
Differential tumour growth between WT and PTGER4 xenograft tumours is not due to enhanced angiogenesis. (**A**) Detection of blood vessels in WT (n = 11 sections) and PTGER4 (n = 11 sections) xenograft tumours as determined by immuno-staining with the endothelial cell marker CD31. (**B**) Quantitative stereology of blood vessel density in WT and PTGER4 xenograft tumours as determined by percentage area positively stained for CD31. (**C**) mRNA expression of the angiogenic factor VEGF in WT (n = 10) and PTGER4 (n = 10) xenograft tumours.

The PTGS-PG pathway has been shown to promote tumorigenesis by enhancing angiogenesis [25], [26], [27]. We investigated whether the enhanced growth observed in PTGER4 tumours compared with WT tumours was a result of increased angiogenesis. We investigated blood vessel density by immunohistochemistry (Figure 3A) and quantitative stereology (Figure 3B) using the endothelial cell marker CD31. We found no difference in immunoreactive staining for CD31 as a percentage area of the tumour in PTGER4 tumours compared with WT tumours (Figure 3B). Since VEGF is the progenitor factor responsible for angiogenesis [28], we investigated whether VEGF levels were altered between the WT and PTGER4 tumours. Similarly, we found no significant difference in VEGF mRNA levels between the two groups, confirming that the enhanced tumour growth in PTGER4 tumours was not a result of increased angiogenesis (Figure 3C).

### Cellular proliferation is enhanced in PTGER4 xenograft tumours

**Figure 4:**
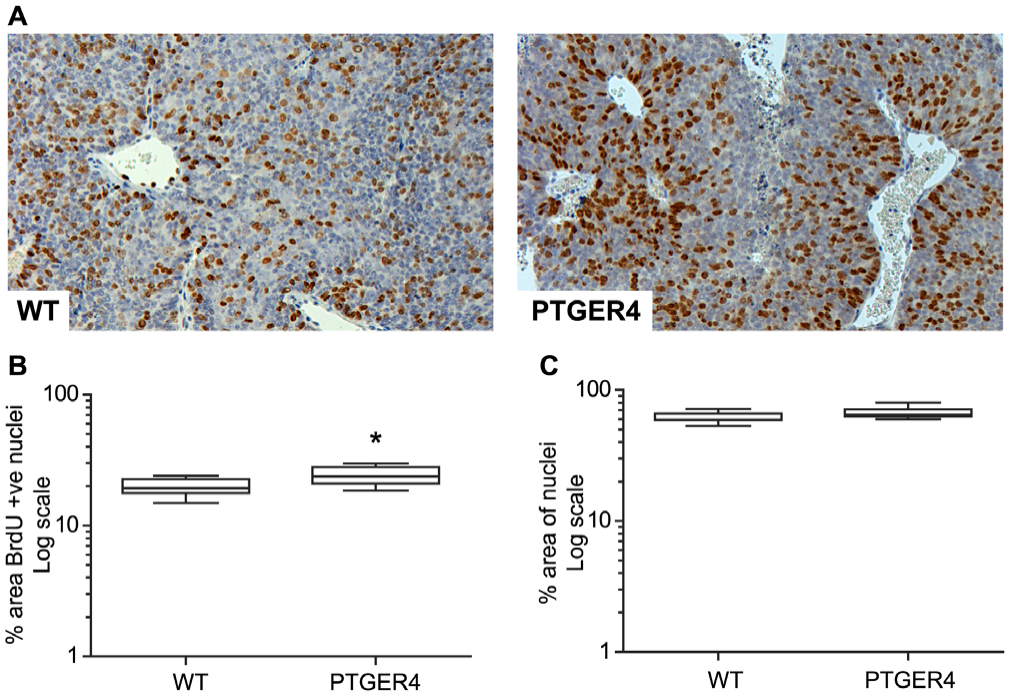
Cellular proliferation is enhanced in PTGER4 xenograft tumours. (**A**) Immunohistochemistry for detection of cellular proliferation in WT (n = 7 sections) and PTGER4 (n = 7 sections) xenograft tumours as determined by staining for BrdU incorporation in the nuclei. (**B**) Quantitative stereology of cellular proliferation in WT and PTGER4 xenograft tumours as determined by percentage area positively stained for BrdU incorporation in the cell nuclei. (**C**) Quantitative stereology of cell density in WT and PTGER4 xenograft tumours as determined by percentage area stained positive for nuclear counterstain. * represents significance at P<0.05.

We assessed whether the rapid increase in growth and volume of the PTGER4 tumours compared to WT tumours was due to an enhanced rate of cellular proliferation of PTGER4 cells *in vivo*. Immunohistochemistry (Figure 4A) and quantitative stereology (Figure 4B) revealed a significant increase in proliferation index in epithelial cells as observed by incorporation of BrdU in PTGER4 tumours compared with WT tumours (Figure 4B, P<0.05). Furthermore we found no alteration in tumour cell density per unit area between PTGER4 tumours and WT tumours (Figure 4C). This suggests that the larger PTGER4 tumours were a result of hyperplasia rather than hypertrophy or non-proliferative tumour expansion.

### PTGER4 xenograft tumours display an increased area of hypoxia

**Figure 5:**
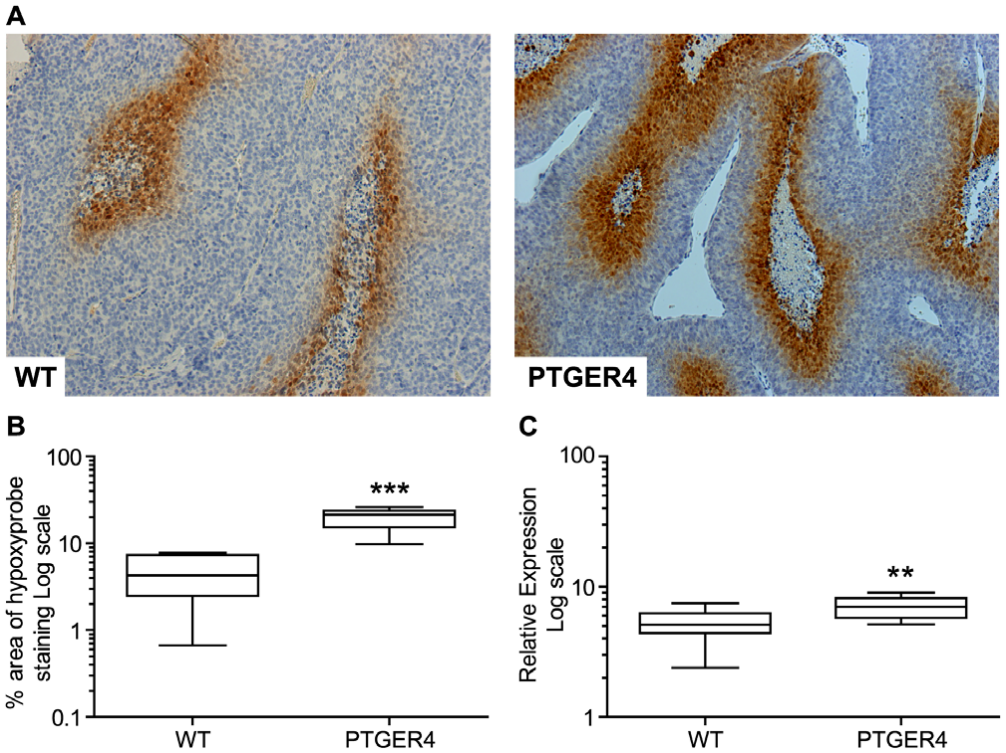
PTGER4 xenograft tumours display increased areas of hypoxia compared with WT control. Immunohistochemistry for detection of hypoxia in WT (n = 5 sections) and PTGER4 (n = 5 sections) xenograft tumours as determined by staining for the Hypoxyprobe pimonidazole. (**B**) Quantitative stereology of hypoxia in WT and PTGER4 xenograft tumours as determined by percentage area stained positive for Hypoxyprobe. (**C**) mRNA expression of the hypoxia inducible factor HIF1α in WT (n = 10) and PTGER4 (n = 10) xenograft tumours. **, *** represents significance at P<0.01 and P<0.001 for PTGER4 versus WT respectively.

Hypoxia is a common recognised driver in many solid tumours and is thought to contribute to development of endometrial adenocarcinoma [29]. We assessed whether the level of hypoxia was altered in the tumours. We found PTGER4 tumours to have increased areas of hypoxia in comparison to WT tumours as indicated by the immunoreactivity for the Hypoxyprobe, pimonidazole, per area of tumour investigated (Figure 5A and 5B, P<0.001). Increased tissue hypoxia in the PTGER4 tumours compared with WT tumours is also supported by quantitative RT-PCR analysis demonstrating higher expression of the hypoxia inducible factor HIF1α (Figure 5C, P<0.01).

### Hypoxia and PGE_2_ via PTGER4 crosstalk to promote cellular proliferation

**Figure 6:**
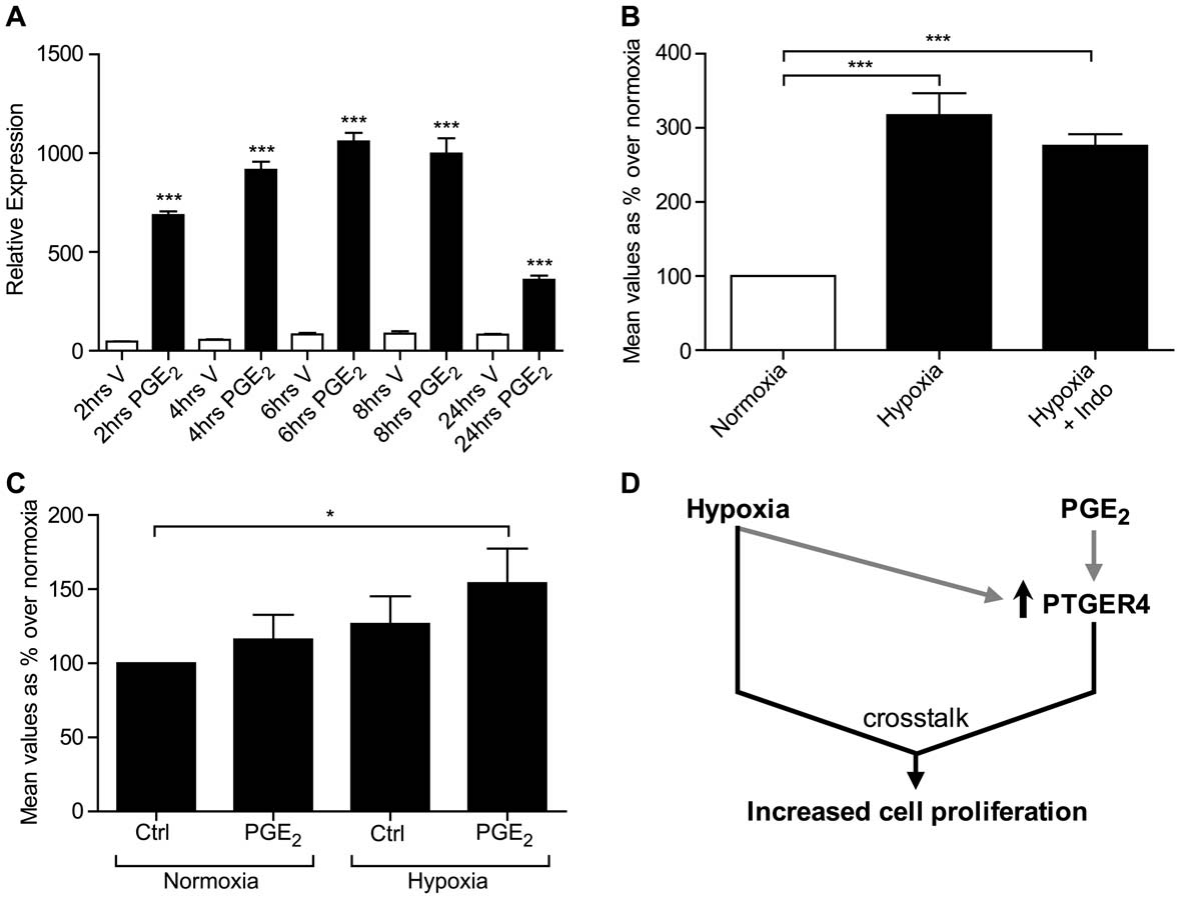
Mechanism of regulation of cellular proliferation in PTGER4 cells in vitro. (**A**) PTGER4 mRNA expression in PTGER4 cells treated with vehicle or 100 nM PGE_2_ for 2, 4, 6, 8 and 24 hours (n = 3). (**B**) PTGER4 expression in PTGER4 cells grown for 24 hours under normoxic and hypoxic conditions, in the absence or presence of the PTGS enzyme inhibitor indomethacin (n = 4). (**C**) Cellular proliferation in PTGER4 cells grown for 24 hours under normoxic (N) or hypoxic (H) conditions in the absence or presence of 1 µM PGE_2_ (n = 4). (**D**) Schematic diagram of findings demonstrating that hypoxia and PGE_2_ both independently elevate PTGER4 expression and synergise to enhance proliferation of PTGER4 cells. *, *** represent significance at P<0.05 and P<0.001 respectively.

Since we found PTGER4 tumours to have an increased proliferation index and hypoxia, we investigated the molecular mechanism regulating PTGER4 expression and proliferation of PTGER4 cells *in vitro* and the contribution of hypoxia to this. We found that treatment of PTGER4 cells with 100 nM PGE_2_ rapidly induced expression of PTGER4 at all time points investigated (Figure 6A, P<0.001). Furthermore, we found that incubation of PTGER4 cells, under hypoxic conditions also induced PTGER4 mRNA expression (Figure 6B, P<0.001). This hypoxia-induced expression of PTGER4 was independent of the PTGS-PG system, as co-incubation of cells with the dual PTGS enzyme inhibitor indomethacin had no effect on PTGER4 expression induced by hypoxia (Figure 6B, P<0.001). These data indicate two independent regulators of PTGER4 expression, the PTGS-PG system and hypoxia induced pathways. Finally we investigated the impact of PGE_2_ signaling and hypoxia on proliferation of PTGER4 cells in vitro. We have shown that neither PGE_2_ nor hypoxia alone can promote proliferation of PTGER4 cells. However, proliferation was significantly elevated following culture of cells with PGE_2_ and hypoxia in combination (Figure 6C, P<0.05).

## Discussion

The PTGS-PG pathway is a potent driver of tumorigenesis in several cancers, including colon, prostate and breast [30], [31], [32], [33]. Expression of PTGS enzymes and biosynthesis of PGE_2_ is elevated in cancers of the female reproductive tract, including endometrial and cervical carcinomas [9], [10], [34]. These findings indicate that PGE_2_, via specific E-series prostanoid receptors may drive the development and progression of reproductive tract cancer in a similar manner to other solid tumours.

We undertook a comprehensive analysis of the expression profile of the PTGES-PGE_2_-PTGER system in normal endometrium across the menstrual cycle and endometrial adenocarcinoma of all grades. As the endometrium of post-menopausal women is no longer under normal hormonal control, the tissue is atrophic and rarely attainable for analysis, we chose normal endometrium as our comparator. We found a variation in the expression of the PTGES-PTGER system across the menstrual cycle, as shown in Figure S1 and is discussed in our recent study [23]. However, of particular significance to this study was the observation of a significant increase in expression of PTGER4 in endometrial cancer compared with normal endometrium across the menstrual cycle. These findings using a substantially larger sample size are consistent with our original observations for expression of these proteins in neoplastic epithelial cells in endometrial cancer [9]. However, more interestingly, within this sample set we found a significant reduction in the expression patterns of PTGER1 and PTGER3 in endometrial adenocarcinomas compared with normal endometrium. These two G protein-coupled receptors differentially couple with Gq and Gi respectively resulting in activation of inositol 1,4,5 trisphosphate and suppression of cAMP [35]. The suppression of these receptors and augmentation of PTGER4, strongly suggest that PGE_2_, produced via PTGS and PTGES2, is a driver of endometrial tumour development via the activation of PTGER4 and initiation of cAMP signalling. Indeed, PTGER4 has been demonstrated to promote human lung and colorectal cancer cell growth [30], [31]. Moreover antagonism of PTGER4 signalling with small molecule receptor antagonists or RNA interference has been shown to inhibit cell growth, proliferation, invasion [33] and metastasis of breast cancer cells [32].

To determine a role for PTGER4 in endometrial tumour development, we stably transfected the full length PTGER4 cDNA transcript into Ishikawa endometrial epithelial cells. We demonstrated translation and functionality of the PTGER4 transgene in Ishikawa cells by immunohistochmistry and cAMP assay and showed that the protein was trafficked to the expected plasma membrane compartment. We investigated the impact of PTGER4 on endometrial tumour development *in vivo* using a nude mouse model. CD1-*Foxn1^nu^* mice were xenografted with WT or PTGER4 cells and xenograft tumour growth was measured over a 35 day period. We found that PTGER4 cells grew at a faster rate resulting in tumours of approximately twice the size compared to WT tumours. Furthermore, higher PTGER4 expression was maintained in PTGER4 tumour xenografts compared with xenografts from WT cells indicating that stability of the transgene was maintained throughout the duration of the experiment. Interestingly, we found that expression of PTGS2 but not PTGS1 was elevated in PTGER4 tumour xenografts compared with WT xenografts. These observations, together with our previous findings showing elevated expression of PTGS2, PGES2 and PGE_2_ in endometrial cancer [9] strongly support a role for PG in driving endometrial tumorigenesis via the PTGER4-cAMP pathway similar to observations in colon cancer cells [30], [36]. Furthermore, other signalling pathways may synergise with or augment PTGER4 signalling *in vivo* to promote the major increase in proliferation we observed in mice xenografts. For example, we have recently shown that multiple PG ligands can converge on the same prostanoid receptor to regulate gene transcription *in vitro* [37]. In addition we have shown that the PTGER2 and PTGFR receptors can crosstalk via the Gs and Gq pathways to enhance cAMP signalling and target gene transcription in endometrial adenocarcinoma cells *in vitro* [38]. It is plausible that similar mechanisms may regulate PTGER4 signalling *in vivo* to promote endometrial tumour cell growth.

Elevated PTGS2 expression and PGE_2_ biosynthesis and signalling has been shown to promote tumorigenesis by enhancing expression of angiogenic factors such as VEGF and increasing angiogenesis [27], [39]. We investigated whether the enhanced growth rate and tumour size in the PTGER4 xenografts compared with WT was due to an enhancement in the elaboration of vascular network to support tumour growth by enhancing nutrient supply and oxygen. However, surprisingly we found no difference in the blood vessel density between the PTGER4 compared with WT tumours as indicated by staining for the endothelial cell marker CD31 or expression of vascular endothelial growth factor. These findings indicate that the observed increase in tumour growth between WT and PTGER4 xenografts was not a consequence of enhanced angiogenesis.

Endometrial cancer growth at the invasive front is regulated by a higher rate of cellular proliferation, driven by the alteration in cell cycle regulation [40], [41]. Although PGE_2_ has been shown to inhibit cellular proliferation in certain cell types [42], blockade of PTGER4 expression inhibits both proliferation and invasion of human inflammatory breast cancer cells [33]. Moreover, alteration in cell cycle regulation and cellular proliferation is known to be regulated in colon cancer cells by the PTGS-PG-PTGER4 pathway [11], [36]. In accordance with these observations, we have found that the elevated growth rate of PTGER4 xenograft tumours in our study is mediated by an increase in the rate of cellular proliferation as indicated by an increase in the incorporation of BrdU in the nuclei of PTGER4 tumour xenografts compared with WT xenografts. Furthermore, we confirmed that the tumour growth was mediated by hyperplasia, rather than hypertrophy or non-proliferative tumour expansion such as enhanced cell migration, since we found no difference in the density of cells in PTGER4 xenograft tumours compared with WT tumours.

Since we found no alteration in angiogenesis, but rather an increase in cellular proliferation, we hypothesised that the PTGER4 xenograft tumours had a higher metabolic demand compared with WT tumours. This would impose higher oxygen and nutrient requirement, which coupled with absence of an increase in angiogenesis, would result in increased hypoxia. Since energy metabolism in cancer cells is regulated in part by hypoxia [43], and hypoxia is a well known driver of tumorigenesis, we investigated levels of hypoxia between the two groups. We found that the PTGER4 xenograft tumours had increased areas of hypoxia as determined by the immunoreactivity for the hypoxyprobe pimonidazole per area of tumour investigated. Moreover we found elevated expression of the hypoxia inducible factor HIF1α in PTGER4 xenografts. Hypoxia inducible factors are well recognised drivers in the tumour microenvironment. They have been shown to induce VEGF expression to promote angiogenesis and tumorigenesis [44]. Furthermore, HIF1α is induced by low oxygen levels and is elevated in fast growing tumours and can activate the inflammatory PTGS-PG system and enhance cellular proliferation [43], [45]. Although we didn't find a correlation with hypoxia and VEGF between the WT and PTGER4 xenografts in our study, it is plausible that hypoxia can induce VEGF equally in both groups early on during tumour growth independent of PTGER4 when vascular networks are forming. Since we didn't find any difference in VEGF expression between WT and PTGER4 xenografts at the time of analysis, it is possible that VEGF regulation by hypoxia in WT and PTGER4 xenografts was already at maximal at the time of analysis in our study.

Our *in vivo* data suggest that endometrial adenocarcinoma growth is regulated by the PTGER4 pathway and hypoxia. To investigate the mechanism and crosstalk whereby PTGER4 and hypoxia could regulate cellular proliferation in endometrial adenocarcinoma cells, we used our PTGER4 stable cell line *in vitro*. We found that PGE_2_ could induce expression of PTGER4, indicating a positive feedback system induced by the PTGS-PGE_2_ synthase-PGE_2_ pathway to maintain PTGER4 expression. Furthermore we found that under conditions of low oxygen tension, PTGER4 expression could similarly be induced independently of the PTGS-PG system. These findings indicate two independent pathways which could drive elevated expression of PTGER4. Perhaps most strikingly, we found that neither pathway alone could significantly induce cellular proliferation, but were required to synergise to promote cellular proliferation as outlined schematically in Figure 6D.

Taken together, our data for the first time highlight a role for PTGER4 in endometrial tumour development. Furthermore our data propose a novel molecular mechanism whereby PGE_2_ biosynthesis, driven by elevated expression of PTGS2 in endometrial adenocarcinoma cells, and hypoxia synergise to increase PTGER4 expression and subsequently enhance cell proliferation and tumour growth.

## Supporting Information

Figure S1 **Expression profile of the PTGES-PTGER system in endometrial adenocarcinoma and normal endometrium.** mRNA expression of PGE_2_ synthase isoforms (PTGES, -2, -3) and E-series prostaglandin receptors (PTGER1–4) in normal endometrium (n = 40; comprising of proliferative n = 10; early secretory n = 10; mid-secretory n = 10 and late secretory n = 10 phase endometrium) and endometrial cancer (n = 29; comprising poorly differentiated n = 10, moderately differentiated n = 10 and well differentiated endometrial adenocarcinoma n = 9). *, **, *** represent significance at P<0.05, P<0.01 and P<0.001.
(TIF)
