# Supplementary figures and images for: Hypoxia and Prostaglandin E Receptor 4 Signalling Pathways Synergise to Promote Endometrial Adenocarcinoma Cell Proliferation and Tumour Growth
Source: PLoS One. 2011 May 12;6(5):e19209. doi: 10.1371/journal.pone.0019209 (PMC3093383; doi:10.1371/journal.pone.0019209)

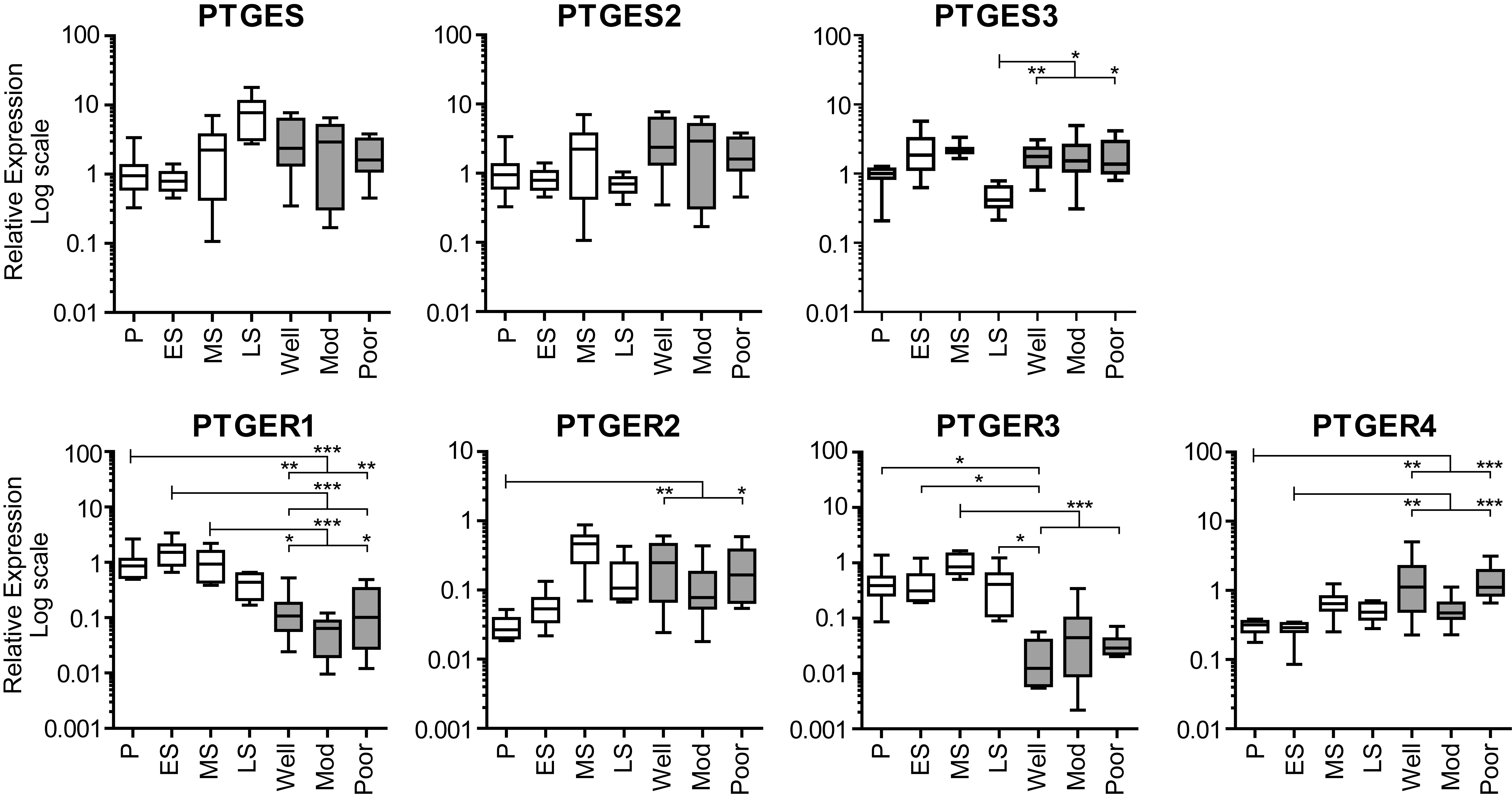

Supplement: Figure S1 — Expression profile of the PTGES-PTGER system in endometrial adenocarcinoma and normal endometrium. mRNA expression of PGE2 synthase isoforms (PTGES, -2, -3) and E-series prostaglandin receptors (PTGER1–4) in normal endometrium (n = 40; comprising of proliferative n = 10; early secretory n = 10; mid-secretory n = 10 and late secretory n = 10 phase endometrium) and endometrial cancer (n = 29; comprising poorly differentiated n = 10, moderately differentiated n = 10 and well differentiated endometrial adenocarcinoma n = 9). *, **, *** represent significance at P<0.05, P<0.01 and P<0.001. (TIF) [file pone.0019209.s001.tif]
